# Clinicians’ Role in the Adoption of an Oncology Decision Support App in Europe and Its Implications for Organizational Practices: Qualitative Case Study

**DOI:** 10.2196/13555

**Published:** 2019-05-03

**Authors:** Christine Jacob, Antonio Sanchez-Vazquez, Chris Ivory

**Affiliations:** 1 Anglia Ruskin University Cambridge United Kingdom; 2 University of Applied Sciences Northwestern Switzerland Brugg Switzerland; 3 Innovation and Management Practice Research Centre Anglia Ruskin University Cambridge United Kingdom

**Keywords:** telemedicine, smartphone, cell phone, oncologists, electronic health record, workflow, workload, workplace, public health practice, technology, perception, health education, mHealth, mobile health, telehealth, eHealth

## Abstract

**Background:**

Despite the existence of adequate technological infrastructure and clearer policies, there are situations where users, mainly physicians, resist mobile health (mHealth) solutions. This is of particular concern, bearing in mind that several studies, both in developed and developing countries, showed that clinicians’ adoption is the most influential factor in such solutions’ success.

**Objective:**

The aim of this study was to focus on understanding clinicians’ roles in the adoption of an oncology decision support app, the factors impacting this adoption, and its implications for organizational and social practices.

**Methods:**

A qualitative case study of a decision support app in oncology, called ONCOassist, was conducted. The data were collected through 17 in-depth interviews with clinicians and nurses in the United Kingdom, Ireland, France, Italy, Spain, and Portugal.

**Results:**

This case demonstrates the affordances and constraints of mHealth technology at the workplace, its implications for the organization of work, and clinicians’ role in its constant development and adoption. The research findings confirmed that factors such as app operation and stability, ease of use, usefulness, cost, and portability play a major role in the adoption decision; however, other social factors such as endorsement, neutrality of the content, attitude toward technology, existing workload, and internal organizational politics are also reported as key determinants of clinicians’ adoption. Interoperability and cultural views of mobile usage at work are the key workflow disadvantages, whereas higher efficiency and performance, sharpened practice, and location flexibility are the main workflow advantages.

**Conclusions:**

Several organizational implications emerged, suggesting the need for some actions such as fostering a work culture that embraces new technologies and the creation of new digital roles for clinicians both on the hospitals or clinics and on the development sides but also more collaboration between health care organizations and digital health providers to enable electronic medical record integration and solving of any interoperability issues. From a theoretical perspective, we also suggest the addition of a fourth step to Leonardi’s methodological guidance that accounts for user engagement; embedding the users in the continuous design and development processes ensures the understanding of user-specific affordances that can then be made more obvious to other users and increase the potential of such tools to go beyond their technological features and have a higher impact on workflow and the organizing process.

## Introduction

### Background

Globally, health care sectors are facing persistent challenges including increasing costs, inconsistent patient care, and a growing burden of chronic disease. One way of overcoming these challenges that have become increasingly the focus of policy change is the transformation of care through a patient-centric service design and taking a more proactive and preventive approach that focuses on quality of life and not only on treating the disease [1].

Smartphones can and do play a strong role here. The latest developments in mobile technology have allowed smartphones to achieve increasingly sophisticated tasks [2], so much so that a new area of health, mobile health (mHealth), has emerged [3].

The global observatory of electronic health in the World Health Organization defines mHealth as “medical and public health practice supported by mobile devices, such as mobile phones, patient monitoring devices, personal digital assistants (PDAs), and other wireless devices” [4]. mHealth solutions differ from other information and communication technology applications in the sense that they are typically user-driven, accessible, and affordable [5]; therefore, a good understanding of the factors impacting user adoption and the roles they play in such adoption are key to the success of such solutions.

However, despite the existence of adequate technological infrastructure and clearer policies, there are situations where users, mainly physicians, resist mHealth solutions [6]. This is particularly relevant, bearing in mind that several studies, both in developed and developing countries, showed that clinicians’ adoption is the most influential factor in such solutions’ success [7-10]. Remarkably, studies show that social aspects are the major factor behind the nonadoption of new technologies, mainly owing to their complexity and the fact that users tend to prefer existing systems over newly introduced ones [11,12]. Furthermore, clinicians’ attitude toward risk is a crucial element in the successful implementation of new health care technologies [13].

### Objectives

Therefore, this research focuses on understanding clinicians’ roles in the adoption of mHealth solutions and its implications for clinicians’ practice. The topic is investigated through the following subquestions:

- What are clinicians’ roles in the adoption of mHealth solutions?

- What are the factors that constrain or afford clinicians’ adoption of mHealth?
- Does the use of mHealth impact social practices in this context? How?

These questions are addressed through a case study of an oncology decision support mHealth solution called ONCOassist and its adoption by clinicians across Europe.

Created in 2012, ONCOassist, shown in (Multimedia Appendix 1), is a free decision support app for oncology clinicians, with over 11,000 active users worldwide in 2019 at the time of writing this paper. The app gives oncologists and oncology nurses easy access to a range of features that aim to help them save time and improve the quality of patient care. The app’s key features are explained in Multimedia Appendix 2.

User research, as a mode of inquiry, has shifted from the assumption that there is a stable material thing (a technology) and separate and identifiable *user preferences* in relation to it toward an understanding in which use and technology are seen as bound together within one another and are constitutive of one another [14-16]. From this ontological perspective, it is challenging to explain *user choices* within existing modes of inquiry [17-20]. It follows that new and more balanced user-research methodologies are needed if questions about user choices and preferences, with regard to new technology, are to be asked.

Contemporary sociomaterial scholars explain that, from their perspective, the social and material combine over time (imbricate) to yield stable sociomaterial constructions—such as stable patterns of use and new organizational structures [21]. Technology partly constrains these constructions, without wholly determining them, as users interact with certain preferred features of technology to create particular *technology affordances* [17,18,22,23]; that is to say that technology is defined not just by its functions and its materiality but by how precisely it is constructed through use. At the same time, use cannot be seen as distinct from those functions or the materiality of the technology in question [22]. Technology *affordance*, in other words, lies somewhere between the user and technology.

Understanding the affordances resulting from this sociomaterial interaction is also key in accounting for how technology impacts on organizations and the process of organizing, as people’s interactions with objects influences the way they organize their microlevel relations [24] and also impacts on the definition of people’s roles [25,26]. Moreover, as organizations become increasingly digital, scholars have portrayed how the technologies used at the workplace are central to the enablement of new forms of organizing and practice and how, in turn, new practices are essential to the enablement of new technologies and new organizational forms [27,28].

Therefore, to better understand the role of clinicians (the users) in the adoption of mHealth (new technologies) and the implications for health care organizations (organizing), the research themes and questions were developed in line with Leonardi’s *Methodological Guidelines for the Study of Materiality and Affordances* to crystallize the focus of the data collected in the interviews [22].

This study used Leonardi’s guidance to build a solid analysis of the role of users in technology adoption and its impact on organizations following these 3 main steps:

- Understanding and documenting the material aspects of technology and their limitations by identifying utility and limitations of the app as perceived by the participating clinicians.
- Linking the material aspects of technology to the tasks that they enable and facilitate by recognizing the real constraints upon opportunities faced by clinicians when using the app.
- Recognizing the processes resulting from these affordances and determining the consequential interactions taking place in the organization by understanding the workflow advantages and disadvantages related to the app’s usage.

The following section explains the research method and how the interview questions and subsequently the analysis stemmed from these 3 steps.

## Methods

A qualitative paradigm was adopted because it gives precedence to *the voices of participants* and the individual and unique *reflexivity of the researcher* [29] and because of the rich insights it offers, which can help draw out and understand clinicians’ experiences and perceptions in different ways that quantitative methods cannot [30,31].

### Data Collection

The data were collected via in-depth semistructured interviews. Given that participants were in many different locations across Europe, not all interviews were held face-to-face, some of them were conducted via Skype, Google Hangouts, or telephone conferencing. Furthermore, physical artifacts such as screenshots of the app, the devices it can be used on, and example written feedback to the developers (eg, app reviews on the app store) were collected to develop a broader perspective about the solution subject of the study [32]. The data collection took place from February 2018 to January 2019, and a total of 17 interviews were conducted with 13 participants (4 were follow-up interviews with the same participants to clarify some details or to ask more questions that were necessary for the analysis). The interviews lasted between 23 min and 110 min and were all conducted and recorded by the first author (CJ) in English. Data collection stopped when an acceptable level of saturation was reached.

### Sampling Techniques and Participants’ Profiles

Purposive sampling was used, where potential participants were selected based on their ability to provide rich and in-depth information about the research topic [29,30]. Key informants in the participating company were contacted, and a snowballing sampling was used to identify suitable participants in their solutions’ user base. The main selection criteria were that participants must be active clinicians or former users of the app being studied. To avoid the possible selection bias that might result from the key informants selectively choosing users with a positive inclination toward the studied solution, it was agreed that participants would be asked if they can in turn refer to other colleagues who were using the solution and were willing to participate. Some participants did refer other colleagues, who were using the app, and some agreed to participate in the study. However, unfortunately, none of the users who had discontinued use of the app agreed to take part in the research when they were asked.

The participants were working in hospitals and clinics in the United Kingdom, Ireland, France, Italy, Spain, and Portugal; and the sample was composed of 8 oncologists (one of them was also an ONCOassist cofounder), 3 nurses, and 2 other members from the ONCOassist team as shown in Table 1.

### Data Analysis and Ethical Considerations

Thematic analysis was used to identify and extract themes that addressed the research questions and explained what each theme could mean as well as the links between themes [33]. QSR’s NVivo was used for coding and then excerpts were selected to create an account that tells the narrative of each theme in a way that helps the reader to understand the analysis. The first author (CJ) conducted the interviews and did the initial analysis and coding; she is a digital strategist with more than 17 years of experience and has contributed to the creation and realization of several digital solutions in health care. Then, the second author (ASV) reviewed the coding; any cases of disagreement about coding were discussed in conjunction with the last author (CI) and mutually agreed. The thematic map is represented in Multimedia Appendix 3, and the phases of the thematic analysis are clarified in Multimedia Appendix 4.

**Table 1:** Sample demographics and characteristics (N=13).

Sample Characteristics		Composition
**Function (%)**		
	8 oncologists (one of them is also an ONCOassist cofounder)	61.5
	3 nurses	23
	2 other ONCOassist cofounders	15.5
**Gender (%)**		
	5 females	38.5
	8 males	61.5
Tech awareness (Participants were asked to define their tech awareness on a scale of 1 to 10), mean		7.25
Health care experience (years), mean		14.5
Mobile health experience (years), mean		7
Location		United Kingdom, Ireland, France, Italy, Spain, and Portugal

The infographic creator, Venngage, was used to visualize some of the findings and not to quantify the data but to emphasize the frequency by showing visually which themes were brought up by more participants than others. The frequency reflected in the infographics counts the theme only once per participant and does not accumulate if the same participant brought up the same theme several times. Such visualization mainly aims to improve the comprehension of the article especially when contrasting 2 elements such as workflow advantages and disadvantages [34] and can provide a clear and simple illustration of the dominant themes and ideas for lay readers [35].

Ethical approval was obtained from the Faculty Research Ethics Panel under the terms of Anglia Ruskin University’s Research Ethics Policy, and all participants were briefed about the research context and signed a study consent form agreeing to participate.

### Interview Themes and Questions

The interview questions addressing each of the 3 following themes are reflected in the interview topic guides available in Multimedia Appendix 5. The first interview guide was used for clinicians and the second was used for providers.

### Theme 1: Accounting for Materials

This theme evolves around understanding the limitations of technology and the types of uses that it enables. Understanding the material aspects of mHealth solutions is crucial because it allows us to identify the different ways it can be used as well as things that cannot be done with it owing to material limitations.

According to Leonardi, technological features “can have various degrees of utility based on the forms into which they are cast” [22]. He explains that a good understanding of the technological features of the solution, recognizing what it can do versus what it cannot do, should help avoid the misconception that users can achieve unlimited tasks with the technologies that they use in their daily work. Therefore, understanding not only the opportunities but also the limitations of mHealth solutions is crucial as a first step of the analysis.

The way a technological tool is built matters because based on that some uses might be very difficult or impossible to achieve, the same way that materials often resist scientists’ efforts to control them, implying that materials have agency [36]. Nevertheless, the user’s ability to rearrange or reshape the materials impacts the way they are used [37].

Therefore, researchers need to understand the materials that form a specific technological tool, how they are organized into specific features, and what such features do or not do. Answering these questions is key to understanding any potential limitations to the use of a specific technology at work; therefore, recognizing the real constraints upon opportunities is the first step toward the rationalization of the role of materiality [22].

### Theme 2: Accounting for Materiality

The focus of this theme is to understand users’ perceptions of technology and how they intend to use it. Leonardi clarifies that people’s views of technology can influence the way they utilize it in their everyday practice; he explains that claiming that a certain technology has materiality means that its materials are being *entangled* or *imbricated* with users’ experiences and culture in forms that make it hard to define the technological tool separately from its context of usage [22]. This understanding acknowledges that users’ intentions and the goals that they want to achieve when using a specific technology have an impact on its affordances.

Several scholars believe that materiality is mainly created through observations of affordance and constraint [18,38,39]; such observations can explain how materiality arises at the junction between technology and its users [40,41]. Some scholars suggest that affordances happen when the existing material properties of a specific technology are given a meaning based on its users’ behavior and that people will not interact with a technology unless they already recognize its utility [42]; this would entail that one technology can result in different outcomes based on the understanding that materiality can offer various affordances [22]. Conversely, other scholars suggest that affordances are created by design and *waiting to be perceived*, are not altered across the diverse contexts of use, and are not created by the users but rather by the designers, and it is up to the users to discover them [43,44].

Hutchby takes the middle ground explaining that affordances go beyond users and technology’s properties and are established based on the kind of relationship formed between people and the technology that they use; accordingly, affordances of a specific solution can change from one context to another even when its material features remain unchanged [45]. This is because users usually have different objectives when approaching materiality, so they distinguish different uses that a specific technology can afford [36]. This relational nature of affordances also means that technology can have various uses, which can result in various ways in how work is organized [46,47].

Therefore, researchers need to understand what social organizations shape the users’ objectives, how these objectives impact users’ understandings of what a specific technology can or cannot do, and what makes users perceive different constraints or affordances based on the options obtainable by the material aspects of the technology itself [22].

### Theme 3: Accounting for Materialization

Leonardi explains that once the researcher understands technology limitations and users’ intentions for its use and how this impacts the affordances, it is important to expand the analysis to comprehend the influence of technology on organizing. This evolves around analyzing and realizing the instances when specific affordances influence and transform the actions, hierarchies, and relationships that constitute the organizing process [22].

It is important to remember that not every technology will clearly impact the organizing process, it is the creation and enablement of specific affordances that helps materials and materiality to materialize in a way that actually impacts organizations and how work is being organized [22]. This idea is emphasized in Leonardi’s example of how a group of technicians who were given a new Information Technology (IT) system started by using it for its initially defined feature of assigning jobs to others, and as they realized its affordances, their use developed to use it for documenting completed jobs, which further evolved to use this past documentation to define who is the best technician to be assigned a new job based on previous experiences. Only then did the new system start to materialize as a crucial element in organizing and optimizing work in that department [48].

Therefore, researchers need to understand how the current patterns of organizing rely on the materiality of specific technologies, why some organizing processes create a social context in which technology can materialize in actions and interactions’ flows, and how have the affordances enabled by technology supported, changed, or transformed the way that people work or interact in a specific organization [22].

## Results

### Accounting for the Materials: Utility and Limitations

Participants were first asked to name the features that they use most in the app to better understand the technological artifacts that they find most useful. They named the features in Table 2.

**Figure 1:**
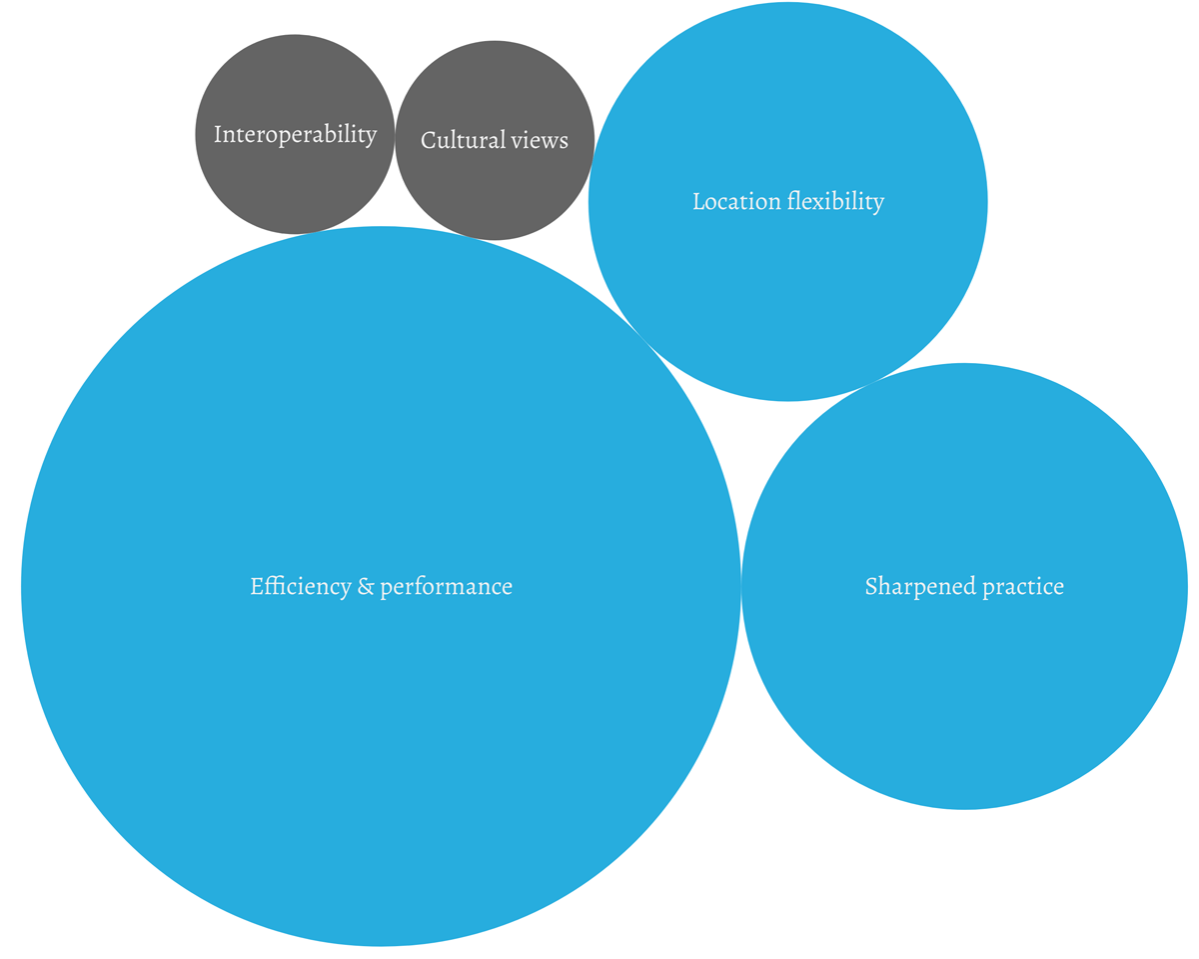
Workflow Improvements and Disadvantages.

They were then asked to explain how the app helped them and their patients on a daily basis to better understand ONCOassist’s utility from their perspective; 3 key themes emerged as visualized in Figure 1. The size of the circles reflects the frequency of the themes; blue represents utility elements and gray, limitations.

All participants mentioned that the app helps them to save time and be more efficient in their daily work. The app also allows a more seamless clinic experience for patients because it enables clinicians to make critical decisions at the point of care, also enhancing patient safety and quality of care. The compact overview of numerous tools and the possibility to switch between the Web application and the mobile app were also highly valued. Sample participants’ quotes are reflected in Multimedia Appendix 6.

**Figure 2:**
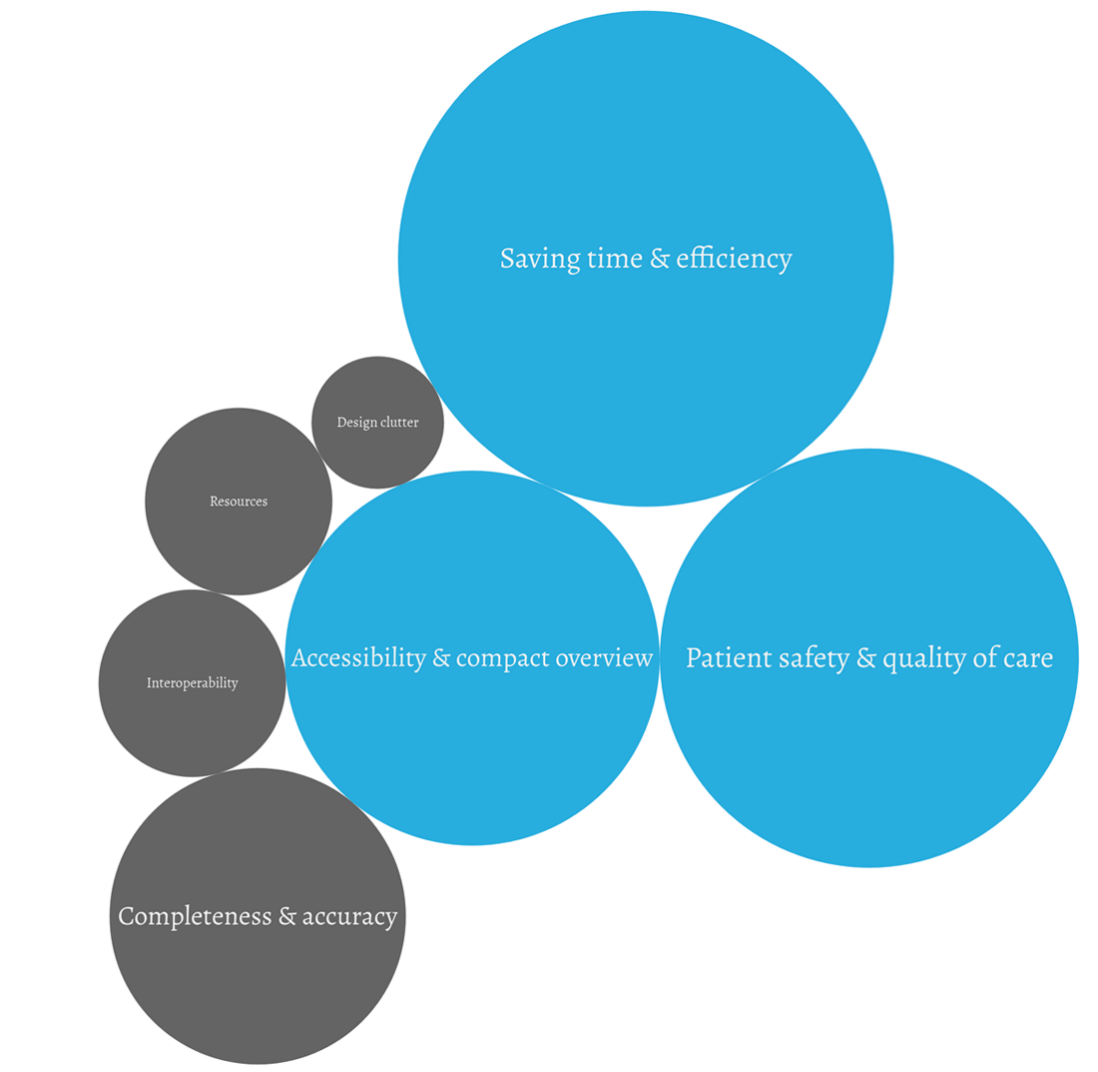
Utility and Limitations.

To complete the picture, the participants were also asked about any limitations they faced when using mHealth apps. And as most participants did not recall many limitations in the ONCOassist app, they were also asked to reflect on limitations associated with other mHealth solutions they had experienced. The limitations are visualized in Figure 2 and explained below.

Factors such as information incompleteness or incorrectness, for example, outdated information, can be perceived as limitations. Apps where the design is too *cluttered* and users cannot find what they are looking for are usually abandoned. This was not the case with ONCOassist, but some participants mentioned it as the reason why they stopped using other apps.

Furthermore, interoperability and electronic medical record (EMR) integration are the key system-related limitations that most mHealth apps are currently facing. Shortage of resources can be an issue, financing is a concern, especially in Europe, as most hospitals are publicly funded. Again, this was not the case with ONCOassist, which is free, but some participants mentioned it as a general barrier with other paid mHealth solutions. Sample participants’ quotes are also reflected in Multimedia Appendix 6.

To have a deeper understanding of perceived limitations in the current features, the participants were also asked to suggest new features that they would like to add to the app. They requested a link to guidelines, adding drug interactions, immunotherapy toxicity, access to new research on different protocols and regimes, adding a prostate-specific antigen (PSA) doubling calculator, a feature for patient monitoring and management, the possibility to use big data for predictive models, breast cancer protocols, clinical trial matching, enabling a community aspect for clinician discussions, local drug prices and reimbursement information, geriatric tool improvement, interpersonalization based on subspecialty, Systemic Anti-Cancer Treatment (SACT) datasets, and product characteristics.

**Table 2:** Most used features.

Feature	Sample quote
Toxicity criteria	“I find it very easy for doses of toxicity… it’s very practical because it’s all in one” (P3)
Calculators	“It’s really useful for when I have to calculate carboplatin dosage using the right formula. It allows me to do it all in the app as opposed to the really, really long calculation that you normally have to make” (P4)
Staging tools	“The main thing that I used the most was the AJCC because it has the super summarized staging” (P7)
Adjuvant tools	“I use ONCOassist for prognostic values in choosing adjuvant chemotherapies” (P6)
Product characteristics	“It’s useful … especially with the new treatments. They’re evolving all the time” (P4)
Customer service	“And the customer service specifically with ONCOassist is very good. I can directly ask, even if I’ve got any issues and I need help with that” (P8)
Drug interaction	“And now the drug interaction checker, which is very, very, very important for us” (P6)
Offline functionality	“A lot of hospitals, the way they are, for some reason the signal is never good. And therefore, that also has a bearing on how these apps work” (P8)

In a follow-up interview, the ONCOassist team explained that features such as the product characteristics and new research on different protocols and regimes already exist in the app but probably the participants who mentioned them were not aware of it. Furthermore, the features drug interactions and PSA doubling calculator were about to be launched at the time of writing of this paper just in time to meet the participants’ requests. The patient monitoring and management feature is also being piloted at the time of writing this paper.

The team explained that other requested features such as drug prices and reimbursement information can be quite challenging to implement because they can vary drastically from country to country and they are subject to frequent change, which requires a large team that is constantly working on updating this information to guarantee it is correct.

### Accounting for Materiality: Constraints and Affordances

To understand affordances, it is important to first start by understanding the intention for use. Therefore, the participants were asked to explain why they decided to use the app in the first place and what they intended to achieve with it.

Most participants decided to use the app because it could help them save time and be more efficient as the information that they need on a daily basis is aggregated reliably in one place; it also increases efficiency by offering a standardized and reliable tool. Recommendations from another oncologist, a medical society, a conference, or even social media channels such as LinkedIn motivated them to try the app; furthermore, usefulness and the constant development and update make the information on the app more accurate than other sources and can improve the quality of care by aiding clinical decisions.

For a more comprehensive analysis, it is also important to consider the reasons for nonadoption; however, it was not possible to recruit nonusers, presumably because they were not interested in the topic. To address this gap, the participants were asked about what they thought could be the reasons why other colleagues might not adopt mHealth solutions. The main reasons given were due to social and cultural factors such as attitude toward technology, age, attitude toward change, previous negative experiences, and perceptions of the use of a personal device at work; furthermore, the nature of the role can also be a factor; depending on how complex the role is, the need for apps may differ. Also, there is a greater awareness of apps that are advertised more and, accordingly, use is more widespread.

The interview questions evolved to address the participants’ more holistic views of the factors impacting the adoption of mHealth from their perspective, going beyond ONCOassist to have a general understanding of barriers and opportunities for mHealth adoption. The main themes are reflected in Multimedia Appendix 7 with sample participants’ quotes and a visual to emphasize their frequency.

App operation and stability including factors such as app size, offline functionality and reliability on connectivity, login issues, and the type of device used may be perceived as constraints or affordances to the use of such apps. Almost all participants mentioned cost as a key factor that could hinder the adoption of such apps, especially for younger clinicians. Most participants mentioned ease of use as a decisive factor for the adoption.

Usefulness is also very important, that the app addresses a need for clinicians to adopt it, purely informative solutions are not perceived as value adding in the form of an app. Endorsement and recommendations from people or institutions that they can trust are an important factor for adoption. Reliability and neutrality of the content, as well as things such as regulatory approval, are decisive factors for the adoption.

Social and cultural factors that impact people’s attitudes toward technology, such as digital savviness or perceptions of the use of a personal device at work, can also play a considerable role in the adoption. Work overload and workplace politics can also play a role in the adoption, depending on what each clinician is focusing on and prioritizing. Finally, the portability and location flexibility of the app as a pocket solution is a significant affordance for adoption.

### Accounting for Materialization: Social and Organizational Implications

The participants acknowledged several social and organizational impacts of the app usage as visualized in Multimedia Appendix 8 to emphasize their frequency, along with participants’ sample quotes.

The app helps users feel more empowered and autonomous. The progress of Health Tech in general is fostering the creation of new digital roles for clinicians and their involvement in digital training and raising awareness about mHealth among their colleagues. The decision to use one mHealth solution or the other lies with the individual clinicians themselves and not centralized through the administration.

Owing to the need for speed, location flexibility, and the limited number of computers available at the workplace, clinicians prefer to reach out to their personal mobile for quick access rather than using the computers at work. Participants also noted that new health technologies, including mHealth, are replacing traditional tools in health care institutions. Also, if systematically embraced by the organization, such an app could be used as a tool for people evaluation and reward.

When asked about workflow improvements, the participants brought up 3 key themes as visualized in Figure 1 and detailed below. The size of the circles reflects the frequency of the themes; the blue color represents workflow advantages, and the gray color represents workflow disadvantages. Sample participants’ quotes are reflected in Multimedia Appendix 9.

The app made their work easier, helped them be more efficient, looked more professional, and meant less interruption. It also sharpened their practice by giving them a backup and a safety net. It offered them location flexibility by helping them overcome computer shortage as well as decision making at the point of care or in meetings; it can also be an alternative in places where a computer is not allowed.

Participants also mentioned 2 workflow disadvantages that should be considered, as shown in Figure 1.

There is still an interoperability issue, as the majority of mHealth apps are not integrated into EMRs and therefore could easily be dismissed in favor of bigger vendors who offer such an integration. Cultural views on the usage of mobile phones at work and the perception that it is *wasting time* can impact mHealth users negatively in the workplace.

### Future Vision and Clinicians’ Role in Adoption

Participants agreed that the future of mHealth is promising and will have a considerable impact on health care. In total, 3 main themes emerged in the discussion about their views of the future of mHealth. They expect a rise in the adoption of mHealth solutions, especially with the standardization of EMRs, and such solutions can then be integrated or plugged into the system. They also expect to be able to benefit from artificial intelligence and all the big data that will result from the implementation of EMRs more widely; such data can be fed through mHealth, for example, to create predictive models and proactive treatment. This could also enable a more patient-centric approach to health care.

They all also agreed that clinicians would play a central role not only in the adoption of mHealth solutions but also their development as shown in Table 3.

**Table 3:** Clinicians’ roles in the adoption of mHealth.

Theme	Sample quote
New digital roles for clinicians, both in digital health start-ups and in health care institutions	“By having CCIOs, clinical information officers, to get involved with the IT side of service provision. So it’s not IT companies just serving up a ready-made product, but they’re actually engaging clinicians at an earlier stage to make them more useful” (P5); “And so we will have more and more, some doctors involved in the development. Maybe if these companies will hire doctors” (P9); “I think of having an experienced nurse that her daily job won’t be to see the patients but rather to see the parameters on her computer … it’s like a control centre that will take care of the patients” (P3)
More involvement in the development and design, not only in the early stages but also through the whole lifecycle of the app.	“Well, hopefully, we'll be engaged in the design of these processors, which we are being, a little bit more than we used to be”, (P11); “I think the physicians who know a lot can have good ideas and more things that can better these apps” (P5)
Education and awareness by acting as ambassadors, raising awareness, and taking the lead for digital health education in health care institutions	“So it’s organising forums that even that they can be discussed, and educating other staff about them” (P12); “Education. Remove the fear. Stigmas about use of mobile phones in workplace, in hospitals need to stop” (P4)
With the efficiencies achieved with mHealth apps, clinicians will be able to focus more on the human side and invest more time with the patients themselves as opposed to spending all the time getting the task done.	“And I don't think there is going to be any machine able to sit with a patient and explain to them and look at them in the eye, all those things that robots don't do but we as humans do” (P7)

### How Do These Insights Make Their Way Back to the Design?

Given the interesting affordances and workflow impacts that emerged from the insights, it was important to understand whether the providers have processes that help them identify these affordances, understand any limitation, and embed these insights in the constant development of the tool. In a follow-up interview with the team to better understand how they engage their users, they explained that their key to success is to follow Steve Bank’s advice by getting out of the building and talking to their users [49]; one of the company’s founders explains, “And part of our CE approval is that we're always engaging with users, always asking how can we improve.”

Therefore, they established a process that enables them to systematically engage with their users depending on how engaged they are as visualized in Multimedia Appendix 10.

All active users receive weekly learning emails for the first few weeks about various features on the app where they are also asked to give feedback. Every user that exceeds 25 active sessions automatically gets an email asking them to fill out a survey; then after 50 sessions, they get another email prompting them to leave app store feedback; they also get emails at various stages throughout their life cycle, asking them what they think, “Is there anything we need to improve?”

Inactive users, defined by the providers as every user that has not used the app for 60 days, automatically get an email asking them to fill out a survey to help the team better understand why these users stopped using the app.

Engaged users, also known as power users, are the users that give regular feedback, they are usually invited to a 30- to 60-min interview during one of the major oncology conferences such as the European Society for Medical Oncology to have a more in-depth discussion with the team and give more detailed feedback. The team explains the importance of these interviews to them:

I think people are more likely to tell you like—when you meet them in person, they’re going to tell you the small little things that annoy them, more so when they’re responding to surveys or emails. The slight little things like, “I have to click here to go back here to find this.” Those kind of things, which I think is really useful.

The team started to identify users who were willing to be more involved to be invited to become official advisors as part of the extended team. One of the founders clarifies, “We need more medical input, so we’re trying to kind of get this panel of clinicians giving us input and paying them for that, in order to kind of help us go to the next level of tools.” This effort has just started, and the team is looking at how they can expand it.

After collecting the feedback, the team discusses together to start screening the users’ input and decide on which features to add accordingly; one of the founders explains:

We are lucky in that the majority of users request similar tools and content, this makes our jobs easier. I think as ONCOassist develops it’s obvious what is missing, and most of our engaged users notice this and point it out. It may be because certain tools and content are becoming more relevant, because of new products on the market. Sometimes we get feedback that may be a little different, which is very useful as well, but 60-70% of the time the requests are similar. Other times we get requests for tools that are difficult for us to build in the short to medium term. We take note of this to see if trends emerge.

The team also ensured that there was a process to allow them to inform the users when the feature they requested was implemented, as they clarify, “We usually take note of the emails, and then when it goes out, we let them know. And there’s also—like we send them a short email, a personalised message, saying, ‘Hey, just so you know, this is now live’. But they’ll get a group email as well,” this helps the team build a personal relationship with these engaged users.

Some of the requests that they receive are also about existing features, as they explain:

Other times—all of what our biggest problem at the moment is around discovery. We have content in there that people don’t know is available, so that’s something we're working on as well. It’s like making sure that people know that we have what they need already… It can be an educational process.

The ongoing challenge that the company is facing, similar to most mHealth start-ups, is monetization, as the team explains:

I feel like we’ve figured out the user interaction, and getting that feedback, and building based on that feedback. Now, for us, the big challenge is figuring out how to kind of have a scalable model with companies that results in us generating good revenues and kind of having on-going relationships in helping and showing improved outcomes.

## Discussion

The results show that technology adoption in our case study is influenced by factors that go beyond the technical and material features of the app; there are other social and organizational factors that play a crucial role in the adoption and success of such new technologies as detailed below.

### Understanding the Possibilities and Limitations of Technological Artifacts

Overall, the participants found the app useful, but not all features were seen as equally so; some were emphasized more than others were and some more relevant to specific cases. Features relevant to everyday tasks at the point of care such us toxicity criteria, calculators, staging tools, and adjuvant tools topped the list. Whereas other features, such as product characteristics, were perceived as important in the case of new drugs because clinicians may not be familiar with new medications and this tool can allow them to quickly check drug-related information, especially given that new drugs are continuously being produced.

The main utility of the app is time saving and efficiency as it helps busy clinicians in their daily work in different ways. From one side, it serves as a memory aid as they cannot memorize all the formulas and checking toxicity calculations, for example, takes time, but with the app, they can get that in a couple of seconds while on the go, this makes it faster and easier to extract information. At the same time, the app helps them ensure higher quality of care and patient safety by enabling them to make critical decisions at the point of care, such as recalculating a dose based on body weight fluctuation, contributing to a seamless patient experience at the clinic or hospital. Clinicians created further affordances by using it as a support tool in their discussions with the patients to better explain to them why some decisions were made (eg, why should they continue with chemotherapy even after the tumor is out) extending the use of the app from a clinician tool to a support tool in patient discussions.

Even though the participants explained that there was nothing in the app that they cannot find elsewhere (eg, medical websites or paper-based calculators), it was the compact overview and the presence of all relevant tools in a single app that is accessible anywhere anytime that made it particularly useful for them. Here, it is clear that the material features of the mobile app, its portability, and accessibility played an important role in the way it is being used by the users and why they perceived it as more convenient and easier to use compared with possible alternatives.

However, the app is not without limitations; the lack of information completeness or correctness is perceived as the main limitation, for example, not all adjuvant tools are on the app; hence, if clinicians need to use one that is missing from the app, they will need to use a different tool to do so. Design-related limitations such as clutter also made it difficult for the users to find what they were looking for; this was not the case for ONCOassist, but the participants mentioned that they did face this issue with other medical apps. Hence, reaffirming Kallinikos’ idea that the way a material feature is shaped or arranged has an impact on the way it is actually used [37]. The lack of EMR integration was also seen as a limitation, such an integration would make it very easy to adjust calculations and toxicity criteria to specific patient profiles and electronic records, taking the app’s utility to a whole new level of convenience and efficiency. This technological interoperability hurdle confirms that often it is the software rather than the feature themselves that can be limiting to some technology uses [22]. Furthermore, there are also social and organizational aspects such as shortage of time and financial resources that can cause limitations to such solutions’ adoption.

When talking about limitations and features that they would like to add to the app, it was interesting to see that some existing features were on the requested list, showing that participants were not always aware of everything that the app could do, which underlined the crucial role of awareness and training. Oudshoorn and Pinch emphasized this point when they explained that the success and adoption of new technologies usually depends on the users’ knowledge of its features and how to use them, stating that “It has long been recognized that the most sophisticated and complex computer hardware and software will come to naught if users don’t know how to use them” [50].

### Understanding Constraints and Affordances as Defined by the Users

When exploring the intention of use and the initial motives that encouraged initial use of the app, participants highlighted several features of the app and what it enabled them to achieve, that is, saving them time, increasing work efficiency, and improving quality of patient care. As Gibson proposes, users might refrain from interacting with an object or in this case a technological solution, without learning what it is good for [42]. However, participants also mentioned some aspects related to what technology in general allows, such as the constant development and update of digital sources, which makes them more accurate than media—such as medical books or paper-based tools. However, social aspects, such as endorsement, also impacted the intention of use, which is a reason for adoption given by nearly all participants. Hence, clinicians mostly trust tools that are endorsed by other clinicians or reliable medical associates they know, showing how social and material aspects start to become entangled, while the decision for adoption does not only rely solely on the tool’s features and capabilities but also on social aspects such as trusted endorsement. This implies that without social aspects such as endorsement some exceptional mHealth solutions might not get used.

Social and cultural factors are perceived as the main source of constraints when it comes to mHealth adoption; general attitude toward technology and change, age, previous negative experiences, and perceptions of the use of a personal device at work can be decisive factors in the nonadoption of mHealth according to participants. For instance, an important example of entanglement of the social and the material is when the use of the smartphone at work is routinely perceived as a nonprofessional activity. Even though professional apps that can run on smartphones are no longer a novelty, the stigma related to the use of personal devices at work persists. Users being concerned with negative perceptions resulting from cultural views of smartphone use at work validates previous scholars’ argument that “culture tells us what something affords,” implying that constraints and affordances are the joint result of the entanglement between the material aspects of technology and cultural practices forming users’ perceptions [22,51].

A further look into the participants’ views of what elements would afford or constrain mHealth helps us understand materiality at the intersection between social and material aspects as Faraj and Azad and Majchrzak et al explain [40,41]. The material aspects mentioned by clinicians mostly evolved around app stability and operation, cost, ease of use, usefulness, and portability; these findings agree with previous research that also reported similar factors [3,52].

However, these aspects intertwined with other important social aspects namely recommendations from people or institutions they can trust, reliability and neutrality of the content (the content provider must be neutral to be trusted, whereas examples given highlighted concerns of bias where content was provided by Pharma companies), social and cultural factors impacting people’s attitudes toward technology, and some organizational factors such as workload and internal politics. These findings complement previous research that shed light on nontechnical factors that play a crucial role in mHealth adoption [53-56].

It’s also worth noting, however, how the material properties of different technologies can also afford different use cases depending on the context they are being used in [22]; this was clear with participants’ experiences around how they switch between the mobile app and the Web app on the desktop when they are using ONCOassist. Several examples showed that the mobile app’s portability, accessibility, and compact overview are the key added values of the solution; however, in specific social contexts, that is, if the clinician is showing a graph to the patients using the app they might prefer to use the Web application to avoid a personal message showing on the phone being seen by the patient, which, in turn, might appear as unprofessional. This highlights the importance of both mobile apps and Web applications as their use can differ from one social context to another.

### Understanding How Technology Materialized in the Organizing Process

Various social and organizational impacts of the app usage were mentioned, namely people empowerment, as the app helps users be more autonomous and gives them more power through knowledge and education; it also empowers young doctors that find themselves in an environment that does not encourage questions as they can find answers to any queries via the app without having to ask a more senior colleague. Furthermore, at this early stage of adoption, the decision making is still individualized, meaning that the decision to use one mHealth solution or another lies with the individual clinicians themselves; however, this may change as the rise of the adoption of such solutions is also driving the creation of new roles for clinicians evolving around digital solutions. We now start to see *clinical information officers* in some hospitals and clinics to deal not only with the medical aspects but also the IT side of such services including topics such as interoperability and software integration, which could lead to more centralized decision making in the future.

Clinicians also prefer to use their personal devices rather than the work devices due to the need for speed, location flexibility, and the limited number of computers available at the workplace. In addition, these apps are rapidly replacing some traditional tools such as textbooks, paper-based calculators, and websites.

Another example that can have clear organizational implications is the use of the app for people evaluation and rewards through, for example, streamlining the calculations and SACT datasets and attaching them to Commissioning for Quality and Innovation, based on which staff members get monetary rewards. However, this is only feasible if it is systematically embraced by the organization and not decided on an individual basis. This reinforces the argument that the impact of the materials and materiality on the organizing process can only be seen if something enables their materialization [22]; in our example, this would not happen unless the management enforces the use of the app for people evaluation and rewards.

As for workflow advantages, the app enhanced users’ efficiency and performance by making their work easier, quicker, more professional, and with fewer interruptions. It also led to a sharpened practice because it provided a backup solution and a safety net to double check drug dosages and calculations then adjust in case of fluctuations in patient’s weight, for example. The personal phone usage also enabled location flexibility; by helping them overcome computer shortages or instant decision making at the point of care or in meetings, it can also provide a solution in places where traditional devices are not permitted such as the clinical room where computers are not allowed because of infection control.

Nevertheless, interoperability and lack of integration with EMRs are seen as key workflow disadvantages because the app as it stands today cannot be directly linked to a specific patient health record, meaning that clinicians have to enter the individual patient values manually for each calculation. Some participants suggest that apps that do not offer EMR integration could easily be dismissed in favor of bigger vendors who offer such an integration. On the contrary, cultural aspects such as assumptions made regarding mobile phone usage in the workplace and the perception that it is a waste of time could impact mHealth users negatively in the workplace unless tackled and awareness is raised.

These insights complement the existing literature that pinpointed the importance of organizational and workflow factors that can considerably hinder mHealth adoption if not addressed properly [6,54,57]

### Accounting for User Engagement: Affordances Enablement

The 3 steps in Leonardi’s methodological guidelines smoothly lead us into understanding the materials, followed by materiality, then materialization, and how the materiality impacts the organizing process. What it does not address though is how can the affordances be enabled through user engagement; in other words, how is user feedback considered and how does this feedback make its way into the design process. It is important to find a way to identify mechanisms that would enable the designers to understand and maximize the potential of the affordances created by the users.

ONCOassist offers a best practice example of embedding the users in the development process and how it made for a constant interaction between the social and the material, allowing the material to develop in a way that best serves both organizational and social practices. They explained how they capture feedback from every user category depending on their level of engagement and that they keep adding new features based on this feedback. They also recently established an advisory board that includes some of the apps’ power users. This enables them to always be on top of newly emerging medical needs and stay relevant in a world that is constantly changing.

From their side, clinicians showed clear interest in being a part of the development process and foresee a rise in digital roles for clinicians, both in digital health start-ups and in health care institutions. This would entail more involvement in the development and design of such solutions, not only in the early stages but also through the whole lifecycle of the app, and they would act as ambassadors raising awareness and taking the lead for digital health education in health care institutions. Some clinicians are clear advocates of such solutions because they believe that it would help them focus more on the human side through the efficiencies that they create.

**Figure 3:**
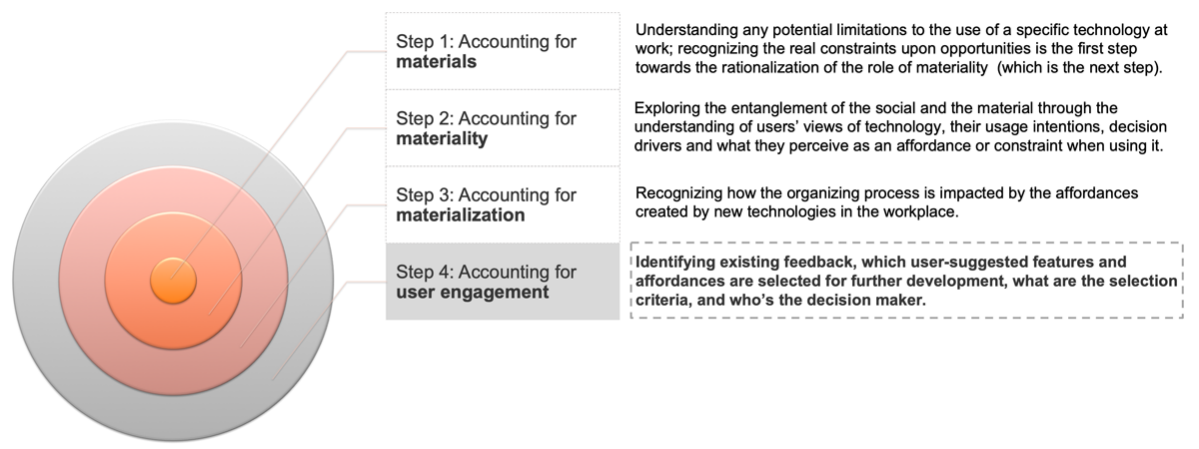
Accounting for User Engagement.

This paper proposes the addition of a fourth step to Leonardi’s guidelines to account for user engagement as visualized in Figure 3 [22], by identifying existing feedback loops, understanding which user-suggested features and affordances are selected for further development, how they are selected and based on which criteria, and who the decision maker is. These questions would help to capture any processes that activate the role of the users and the implementation of affordances that might arise.

Including the users in the constant development and testing of innovative tools is not without challenges, especially considering the very competitive environment that mHealth start-ups are operating in; hence, they are protective of their ideas, which results in most of the design and testing being done inside the company without involving anyone external [58]. This, however, needs to change to enable the user-created affordances to make their way to the design and to ensure that these new tools will stay relevant to the users in a world that changes continuously. A balance between user engagement and confidentiality can be achieved through advisory contracts, nondisclosure agreements, or the like.

### Practical and Theoretical Implications

This case demonstrates the affordances and constraints of mHealth technology at the workplace, their implications for the organization of work, and clinicians’ role in their constant development and adoption. The research findings confirmed that factors such as app operation and stability, ease of use, usefulness, cost, and portability play a major role in the adoption. However, other social factors such as endorsement, neutrality of the content, attitude toward technology, workload, and internal politics are also perceived as key determinants of adoption.

Several organizational implications also emerged, suggesting the need for some actions such as fostering a work culture that embraces new technologies and the creation of new digital roles for clinicians both on the hospitals or clinics and on the development sides but also more collaboration between health care organizations and digital health providers to enable EMR integration and solve any interoperability issues.

From a theoretical perspective, this study suggests the addition of a fourth step to Leonardi’s methodological guidance to account for user engagement; embedding the users in the continuous design and development processes ensures the understanding of user-specific affordances that can then be made more obvious to other users and increase the potential of such tools to go beyond their technological features and have a higher impact on workflow and the organizing process.

### Limitations and Recommendations for Future Research

This case study is limited to a specific mHealth solution and a specific geography during a specific timeframe, and therefore its findings may not be generalizable to other contexts where, for example, the health care system and its regulations could be considerably different. Furthermore, the sample size is relatively small and excluded nonusers because their recruitment proved to be very challenging. Moreover, given the constantly evolving nature of mHealth, the context of the research might change very quickly necessitating new research to update the findings.

To address some of these limitations, future research should cover other mHealth solutions in other geographies, timeframes, and contexts. It would also be very relevant to include some nonusers to the participants mix to cover their views as well.

## Appendix

Multimedia Appendix 1 ONCOassist, a decision support app for oncologists.

Multimedia Appendix 2 ONCOassist’s key features at the time of writing this paper.

Multimedia Appendix 3 NVivo code scheme.

Multimedia Appendix 4 Phases of thematic analysis after Braun and Clarke.

Multimedia Appendix 5 Interview guides for users and providers.

Multimedia Appendix 6 Utility and limitations.

Multimedia Appendix 7 Barriers and opportunities.

Multimedia Appendix 8 Social and organizational impacts.

Multimedia Appendix 9 Workflow improvements and disadvantages.

Multimedia Appendix 10 ONCOassist's user engagement strategy.

## Abbreviations

EMR: Electronic Medical Record
IT: Information Technology
mHealth: mobile health
PSA: prostate-specific antigen
SACT: Systemic Anti-Cancer Treatment
