# Supplementary figures and images for: Clinicians’ Role in the Adoption of an Oncology Decision Support App in Europe and Its Implications for Organizational Practices: Qualitative Case Study
Source: JMIR Mhealth Uhealth. 2019 May 3;7(5):e13555. doi: 10.2196/13555 (PMC6524456; doi:10.2196/13555)

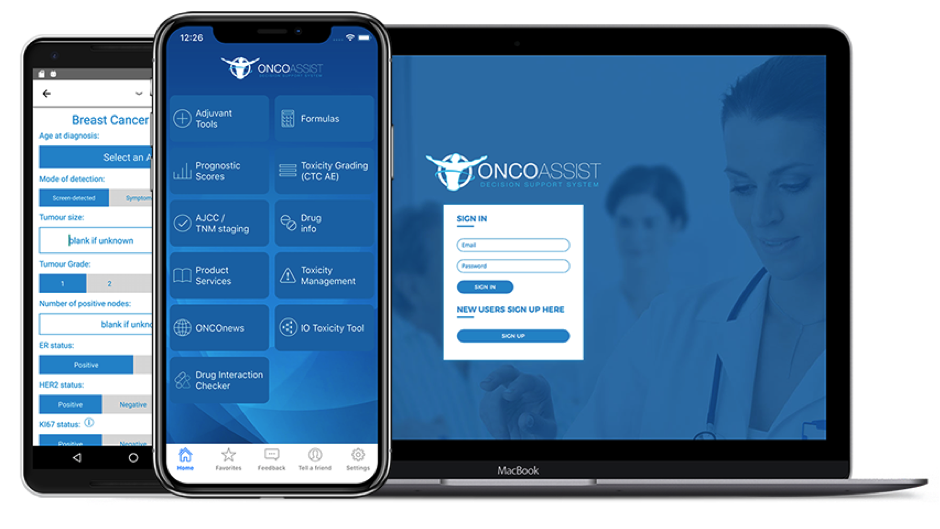

Supplement: Multimedia Appendix 1 [file mhealth_v7i5e13555_app1.png]

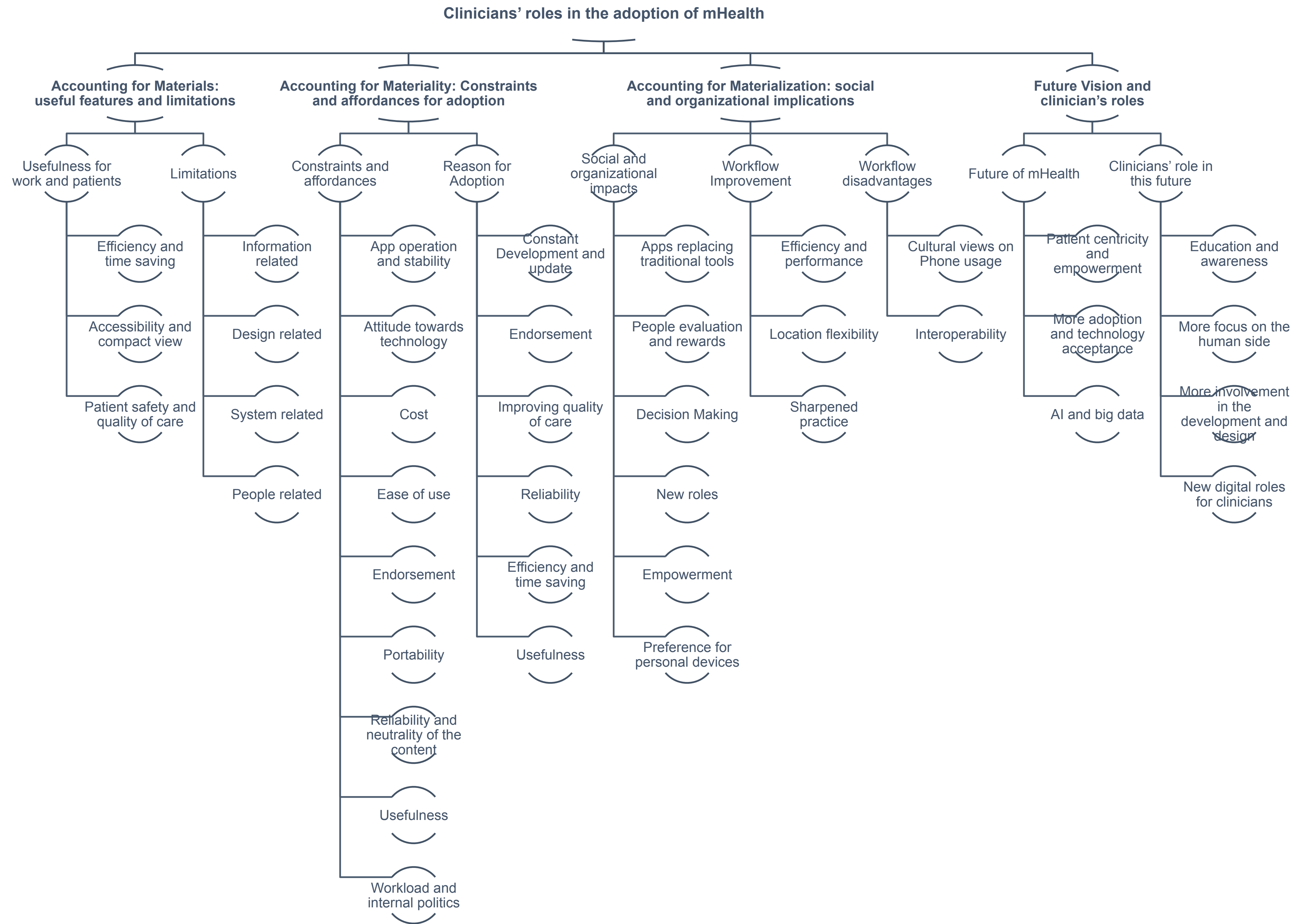

Supplement: Multimedia Appendix 3 [file mhealth_v7i5e13555_app3.pdf]

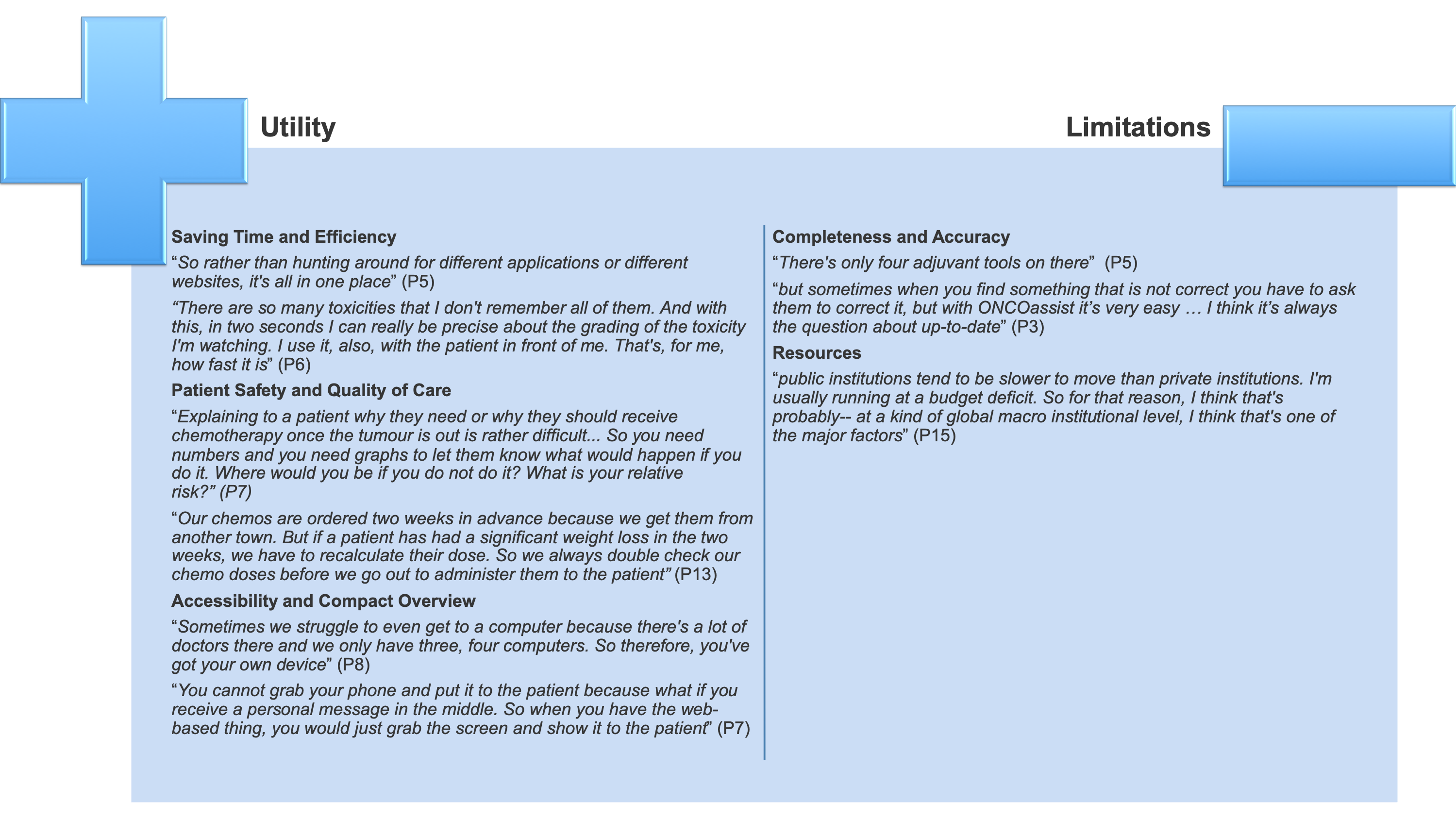

Supplement: Multimedia Appendix 6 [file mhealth_v7i5e13555_app6.png]

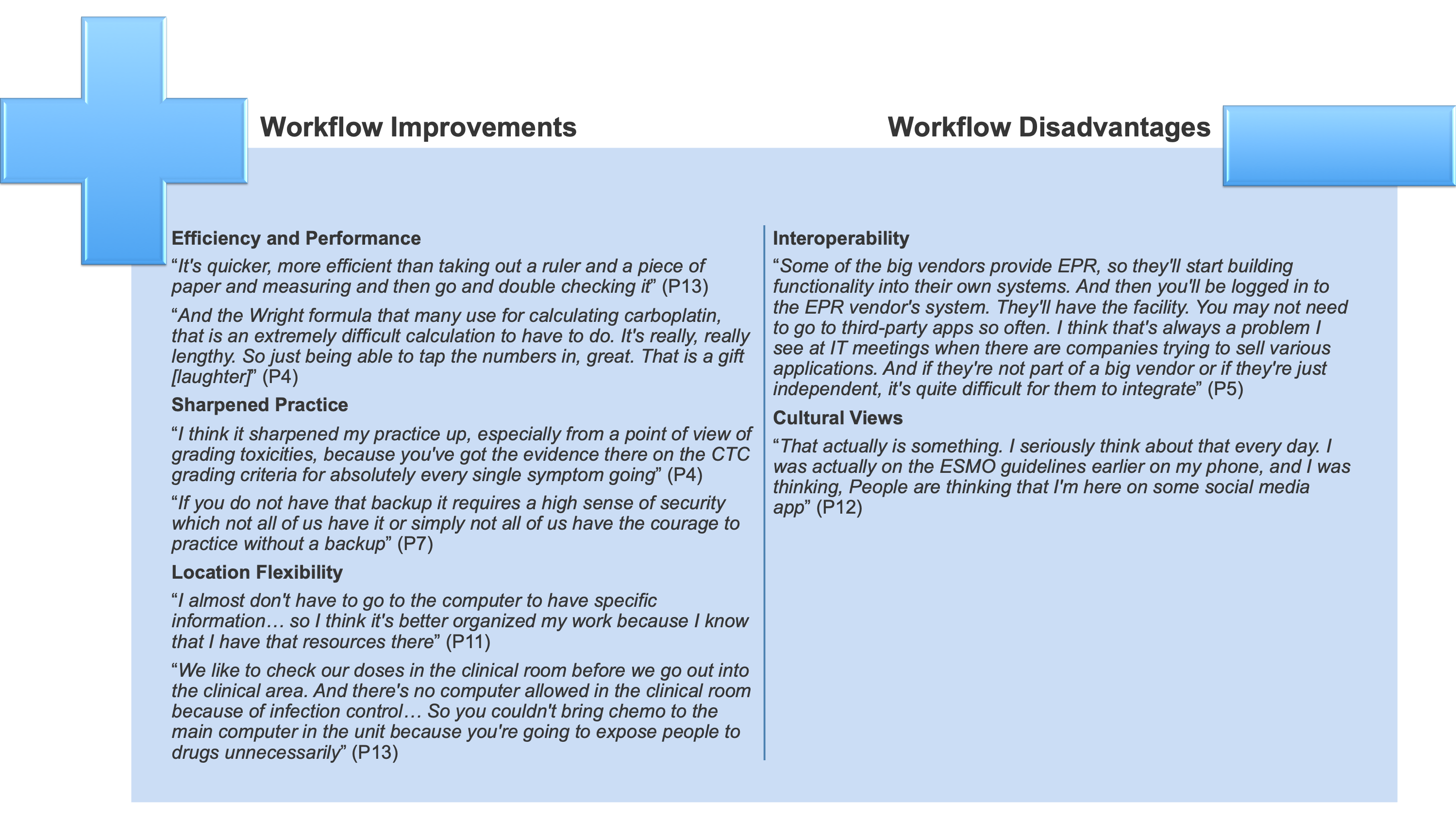

Supplement: Multimedia Appendix 9 [file mhealth_v7i5e13555_app9.png]

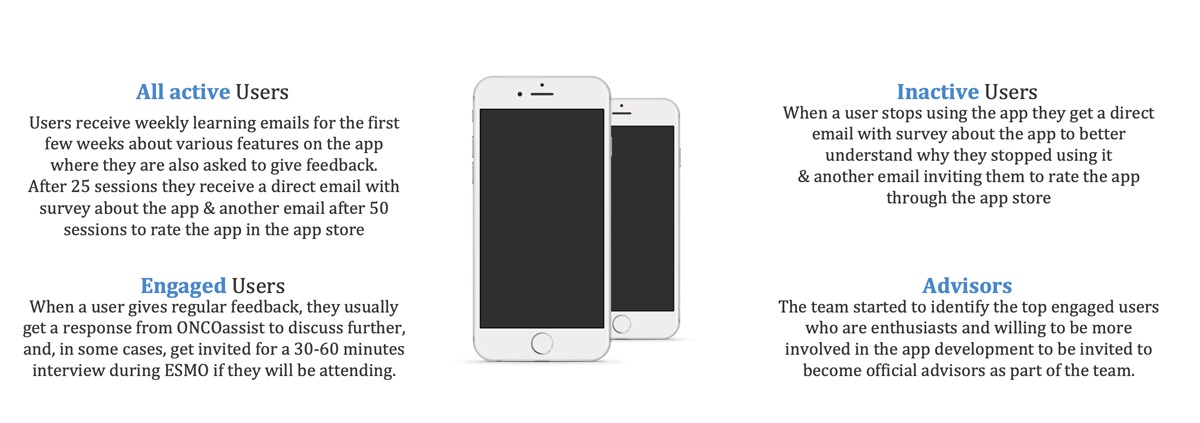

Supplement: Multimedia Appendix 10 [file mhealth_v7i5e13555_app10.png]
