# Supplementary material for: Clinicians’ Role in the Adoption of an Oncology Decision Support App in Europe and Its Implications for Organizational Practices: Qualitative Case Study
Source: JMIR Mhealth Uhealth. 2019 May 3;7(5):e13555. doi: 10.2196/13555 (PMC6524456; doi:10.2196/13555)
Supplement: Multimedia Appendix 2 [file mhealth_v7i5e13555_app2.pdf]

ONCOassist's key features at the time of writing this paper

- **Adjuvant tools:** can be used to get a 5 and 10-year overall survival of patients with and without chemotherapy in an adjuvant setting, this helps them inform patients as to why they may or may not be prescribing chemotherapy (e.g. Prediction algorithm estimating survival rates for breast/lung/colon/GIST cancer based upon risk factors and treatment).

- **Formulas:** over 20 interactive formulas including offline access to enable clinicians to make the necessary calculations at point of care (e.g. body surface area / chemotherapy dose calculator to adjust the chemo dosage if a patient loses or gains weight since the last prescribed dosage).

- **Prognostic scores:** over 14 prognostic scores enabling clinicians to get the scores they are looking for based on a few questions and patient characteristics (e.g. predicting survival in patients with metastatic renal cell carcinoma).

- **Common Toxicity Criteria:** a set of criteria for the standardized classification of adverse effects of drugs used in cancer therapy.

- **AJCC TNM Staging:** enables an easy and quick feature to help clinicians in their cancer reporting and classification, e.g. Tumor size, Lymph Nodes affected, Metastases.

- **Drug Info:** gives users access to a comprehensive list of oncology specific drugs information allowing them to jump in and out of specific sections quickly.

- **Drug Interaction checker:** enables users to quickly search combinations of drug interactions to identify if they are safe to use.

- **ONCOnews:** allows easy access to most up to date news and information in the field of oncology, personalized based on the user's specialist interests.
