# Supplementary material for: Clinicians’ Role in the Adoption of an Oncology Decision Support App in Europe and Its Implications for Organizational Practices: Qualitative Case Study
Source: JMIR Mhealth Uhealth. 2019 May 3;7(5):e13555. doi: 10.2196/13555 (PMC6524456; doi:10.2196/13555)
Supplement: Multimedia Appendix 4 [file mhealth_v7i5e13555_app4.pdf]

Phases of thematic analysis after Braun & Clarke

**Familiarizing yourself with your data**	Transcribing data, reading and re-reading the data, noting down initial ideas.
**Generating initial codes**	Coding interesting features of the data in a systematic fashion across the entire data set, collating data relevant to each code.
**Searching for themes**	Collating codes into potential themes, gathering all data relevant to each potential theme.
**Reviewing themes**	Checking if the themes work in relation to the coded extracts (Level 1) and the entire data set (Level 2), generating a thematic 'map' of the analysis.
**Defining and naming themes**	On-going analysis to refine the specifics of each theme, and the overall story the analysis tells, generating clear definitions and names for each theme.
**Linking themes to explanatory frameworks, models and concepts**	Making a contribution to theory, reflecting on the validity of different sociomaterial approaches. Building new approaches and theoretical categories and concepts.
**Producing the report**	The final opportunity for analysis. Selection of a vivid, compelling extract examples, final analysis of selected extracts, relating back of the analysis to the research question and literature, producing a scholarly report of the analysis.

Source: After (Braun & Clarke, 2006, S. 87).
