# Supplementary material for: Clinicians’ Role in the Adoption of an Oncology Decision Support App in Europe and Its Implications for Organizational Practices: Qualitative Case Study
Source: JMIR Mhealth Uhealth. 2019 May 3;7(5):e13555. doi: 10.2196/13555 (PMC6524456; doi:10.2196/13555)
Supplement: Multimedia Appendix 5 [file mhealth_v7i5e13555_app5.pdf]

Interview guide - Clinicians

Background Questions

1. Participant introduction
   - Tell me about your role in the organization
   - How long have you worked in healthcare?
   - How long have you been using Mobile Health?
   - How would you define your level of technical awareness

2. How would you define "Mobile Health" in one sentence?

Theme 1: "Accounting for materials"

3. Tell me about the mHealth Solution(s) that you are using
   - What are its main features?
   - Are there any limitations in its features?
   - If you would add one feature what would it be?

4. How did it help you and your patients?

Theme 2: "Accounting for materiality"

5. Tell me about what you wanted to achieve when you decided to use mHealth

6. What were the factors that influenced your decision to adopt mHealth?
   - Which would you consider a barrier and which an opportunity?

7. Who made the decision to implement mHealth?
   - How widespread is its use?
   - What do your colleagues think?
   - Do you recommend it to others?

Theme 3: "Accounting for materialization"

8. What influence did mHealth have on your work/the work of others?
   - Did it improve it?
   - Was the previous practice better for some things?

9. Have these solutions led to changes in how the organization works, its rules or the use of other tools / technologies?

10. How have the uses of these solutions sustained, altered, or transformed the way that people interact in your organization?

11. In your opinion, what does the future hold for mHealth? And what roles will HCPs play in shaping this future?

Interview guide - Providers

Background Questions

1. Participant introduction
   - Tell me about your role in the organization
   - How long have you worked in healthcare (or Healthcare Tech)?
   - How long have you been working on Mobile Health apps?
   - How would you define your level of technical awareness

2. How would you define "Mobile Health" in one sentence?

Theme 1: "Accounting for materials"

3. Tell me about your mHealth Solution
   - What are its main features? What do the features do or not do?
   - Are there any limitations in the features?
   - If you would add one feature, what would it be?

4. How is it intended to help HCPs/patients?

Theme 2: "Accounting for materiality"

5. Tell me about what you wanted to achieve when you decided to create your mHealth app

6. Based on your experience with your customers: What are the factors that influence HCPs' decision to adopt mHealth?
   - Which would you consider a barrier and which an opportunity?

7. In your experience: Who usually makes the decision to implement mHealth?
   - How widespread is its use?
   - What do HCPs think?

Theme 3: "Accounting for materialization"

8. What do you think is the influence of your mHealth app on HCPs' daily work?
   - Did it improve it?
   - Was the previous practice better for some things?

9. Have your mHealth app led – or could it potentially lead - to any changes in how healthcare organizations work, their rules, or the use of other tools / technologies?

10. How have – or potentially could - the use of your mHealth app sustained, altered, or transformed the way that people interact in healthcare organizations?

11. In your opinion, what does the future hold for mHealth? And what will be the role(s) of HCPs in this development?
