# Supplementary material for: Clinicians’ Role in the Adoption of an Oncology Decision Support App in Europe and Its Implications for Organizational Practices: Qualitative Case Study
Source: JMIR Mhealth Uhealth. 2019 May 3;7(5):e13555. doi: 10.2196/13555 (PMC6524456; doi:10.2196/13555)
Supplement: Multimedia Appendix 7 [file mhealth_v7i5e13555_app7.pdf]

Barriers and Opportunities

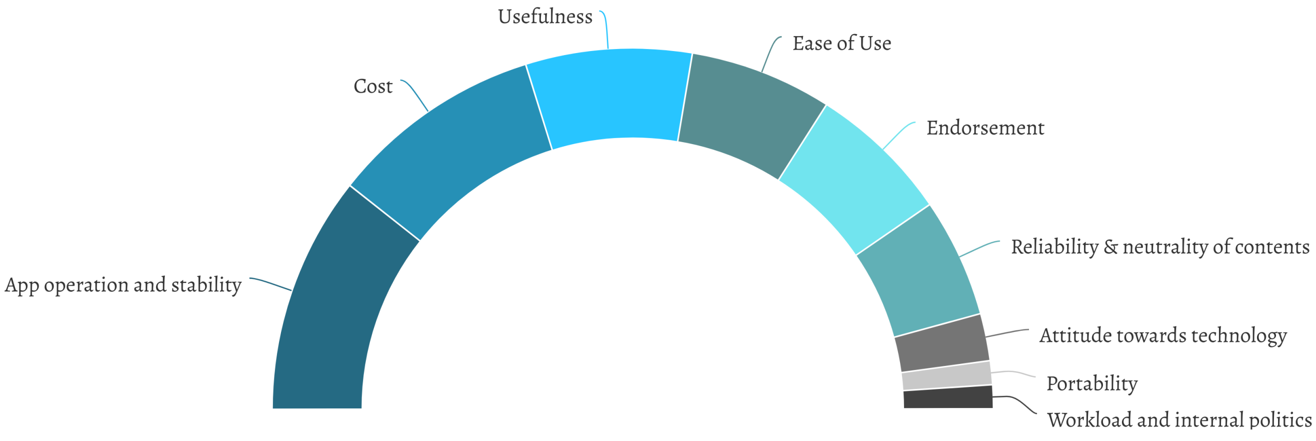

Sample Participants' Quotes

Theme	Sample Quotes
App operation	*"Some of the apps you use slow down your mobile or they're always running in the background and therefore they use up a lot of battery. So that again is not something that you want to happen" (P8)*
Cost	*"When you're in training and you're in initial years, at that point, definitely if it's so expensive then you wouldn't be using it"* (P8)
Usefulness	*"I didn't want it using up space on my phone when I wasn't using it enough. They don't give calculations or formulas. They're only giving you information"* (P 13)
Ease of Use	*"I've often downloaded lots of apps and deleted them. But once I download that and see how easy it is to access, it stayed on my phone"* (P 12)
Endorsement	*"So I would say the encouragement from ESMO is definitely a factor because probably this endorsement in itself has its strength"* (P8)<br><br>*"I went for me on to Young Oncologists Facebook group and everybody was talking about it"* (P9)
Reliability and Neutrality	*"They regularly keep their apps updated. And if you realize that somebody doesn't, then you quit using that app and move to the next one"* (P7)
Social and Cultural factors	*"But I know people, even people from my age that are somewhat challenged regarding apps in general. So I don't think they would obtain the benefit I do"* (P7)<br>*"When you are seen to be on your telephone at work, people automatically assume that you're sending a text message or that you're looking at Facebook or Twitter or something when actually, you're using it to help you make decisions. So for me, there's still that barrier there, that stigma attached to using your telephone at work, and it needs to go"* (P4)
