# Supplementary material for: Clinicians’ Role in the Adoption of an Oncology Decision Support App in Europe and Its Implications for Organizational Practices: Qualitative Case Study
Source: JMIR Mhealth Uhealth. 2019 May 3;7(5):e13555. doi: 10.2196/13555 (PMC6524456; doi:10.2196/13555)
Supplement: Multimedia Appendix 8 [file mhealth_v7i5e13555_app8.pdf]

Social and Organizational Impacts

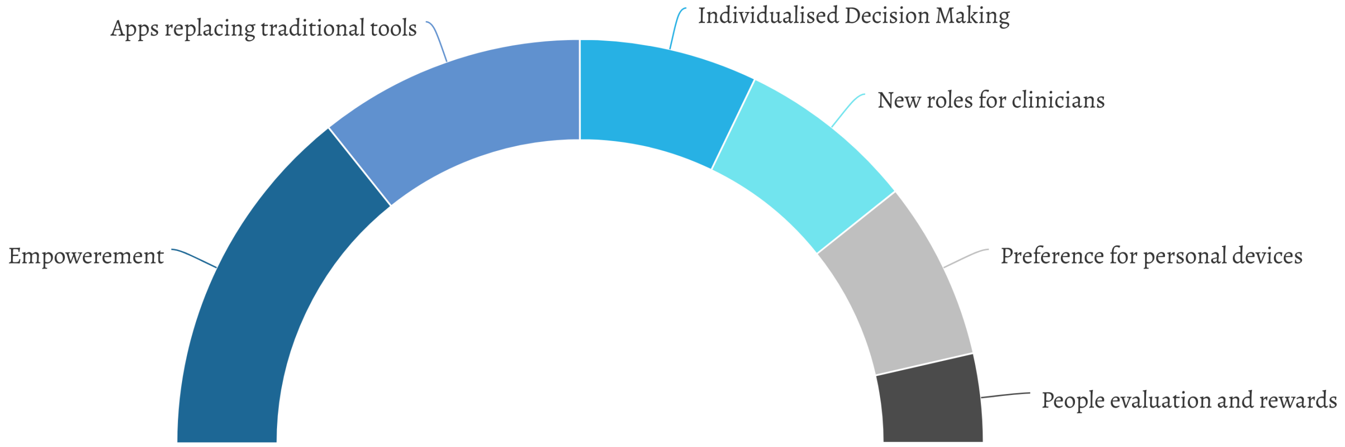

Sample Participants' Quotes

Theme	Sample Quotes
Empowerment	*"Because education and knowledge equals power at the end of the day… it means you can be more autonomous"* (P 4)<br>*"If there are some institutes where there is a bit of hostility, to be very honest, and you're not encouraged-or yourself, you're made to feel that if you ask too many questions, that you're the one who's not knowledgeable, and therefore, you don't ask too many questions"* (P 8)
New roles for Clinicians	*"Having … CCIOs, clinical information officers, to get involved with the IT side of service provision"* (P5)
Individualized decision making	*"It is very much individualized, there's no set thing"* (P12)<br>*"At an individual level, things like apps and things like that are different, and the individual makes a decision on that themselves"* (P15)
Preference for personal devices	*"But I would often look up the common toxicity criteria as well, if I have my phone in my pocket. I wouldn't always have access to an iPad or something, if I was on the ward. So that gives me quick access"* (P12)
Apps replacing traditional tools	*"When I came here first, all we had was a card that had weights on one side, heights on the other side, and you had to bring a ruler between the two to calculate"* (P13)<br>*"Well, books are practically gone... since there's so many changes, we mostly based our knowledge in online sources"* (P7)
People Evaluation	*"With chemotherapy, we have something called the SACT datasets that we have to comply with. We have to record certain elements, and they've attached CQUINS to this dataset now whereby we get money if we comply… And if we fulfil all the criteria, then you get money or you don't get fined. So I think it would be so much easier if we've got mobile technology that we could just type in data that we needed to type in in order to meet the essential criteria"* (P4)
